# Supplementary material for: Bridging the gap between presynaptic hair cell function and neural sound encoding
Source: eLife. 2024 Dec 24;12:RP93749. doi: 10.7554/eLife.93749 (PMC11668530; doi:10.7554/eLife.93749)
Supplement: Supplementary file 1. [file elife-93749-supp1.docx]

**Supplementary Table 1.** Spontaneous activity (*SR*) was calculated from time windows without stimulation with the IHC held at -58 mV (Total time for SR calculation). This total time was calculated from the cumulative recording time of either from 5 – 10 s recordings and/or from the segments before and after a depolarizing pulse.

| Cellname | Side | SR | Total time for SR calculation | Cumulative time of 5-10 s recordings (s) | Cumulative time before/after a depolarizing pulse (s) |
| --- | --- | --- | --- | --- | --- |
| l140817_1 | Modiolar | 1.07 | 36 |  | 36 |
| l140817_2 | Modiolar | 0.38 | 28.8 |  | 28.8 |
| l140817_3 | Modiolar | 0.05 | 43.2 |  | 43.2 |
| l300817_1 | Modiolar | 0.00 | 31.2 |  | 31.2 |
| l240518_1 | Pillar | 0.22 | 91.2 |  | 91.2 |
| l180618_1 | Pillar | 1.00 | 126.8 |  | 126.8 |
| l031220_1 | Pillar | 0.00 | 10 | 10 |  |
| l041220_1 | Pillar | 0.00 | 10 | 10 |  |
| l280817_1 | Modiolar | 0.00 | 3.69 |  | 3.69 |
| l200418_1 | Pillar | 3.92 | 3.06 |  | 3.06 |
| l200618_1 | Pillar | 0.20 | 20.8 |  | 20.8 |
| l260618_1 | Pillar | 2.05 | 20.8 |  | 20.8 |
| l090718_2 | Pillar | 4.28 | 56.8 |  | 56.8 |
| l170718_1 | Pillar | 0.38 | 23.2 |  | 23.2 |
| l240718_4 | Pillar | 17.93 | 7.2 |  | 7.2 |
| l250718_1 | Pillar | 3.18 | 38.4 |  | 38.4 |
| l270718_1 | Pillar | 9.00 | 20 |  | 20 |
| l090119_1 | Modiolar | 0.00 | 20 | 20 |  |
| l150618_1 | Pillar | 1.49 | 12 |  | 12 |
| l290321_1 | Modiolar | 0.03 | 38 | 10 | 28 |
| l300321_1 | Modiolar | 0.36 | 85.2 | 50 | 35.2 |
| l120421_1 | Pillar | 0.21 | 35.6 | 10 | 25.6 |
| l130421_1 | Pillar | 0.00 | 35.4 | 25 | 10.4 |
| l301118_1 | Modiolar | 0.15 | 40 | 40 |  |
| l170119_1 | Pillar | 0.80 | 10 | 10 |  |
| l090419_1 | Modiolar | 0.00 | 10 | 10 |  |
| l120419_1 | Modiolar | 0.70 | 10 | 10 |  |
| l230920_2 | Pillar | 0.00 | 15 | 15 |  |
| l040221_1 | Pillar | 0.20 | 10 | 10 |  |
| l230522_1 | Pillar | 1.26 | 49.6 | 10 | 39.6 |
| l230817_2 | Modiolar | 0.16 | 6.12 |  | 6.12 |
| l161017_1 | Modiolar | 0.00 | 2.34 |  | 2.34 |
| l200522_2 | Pillar | 0.00 | 40 | 40 |  |
